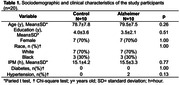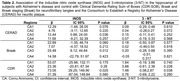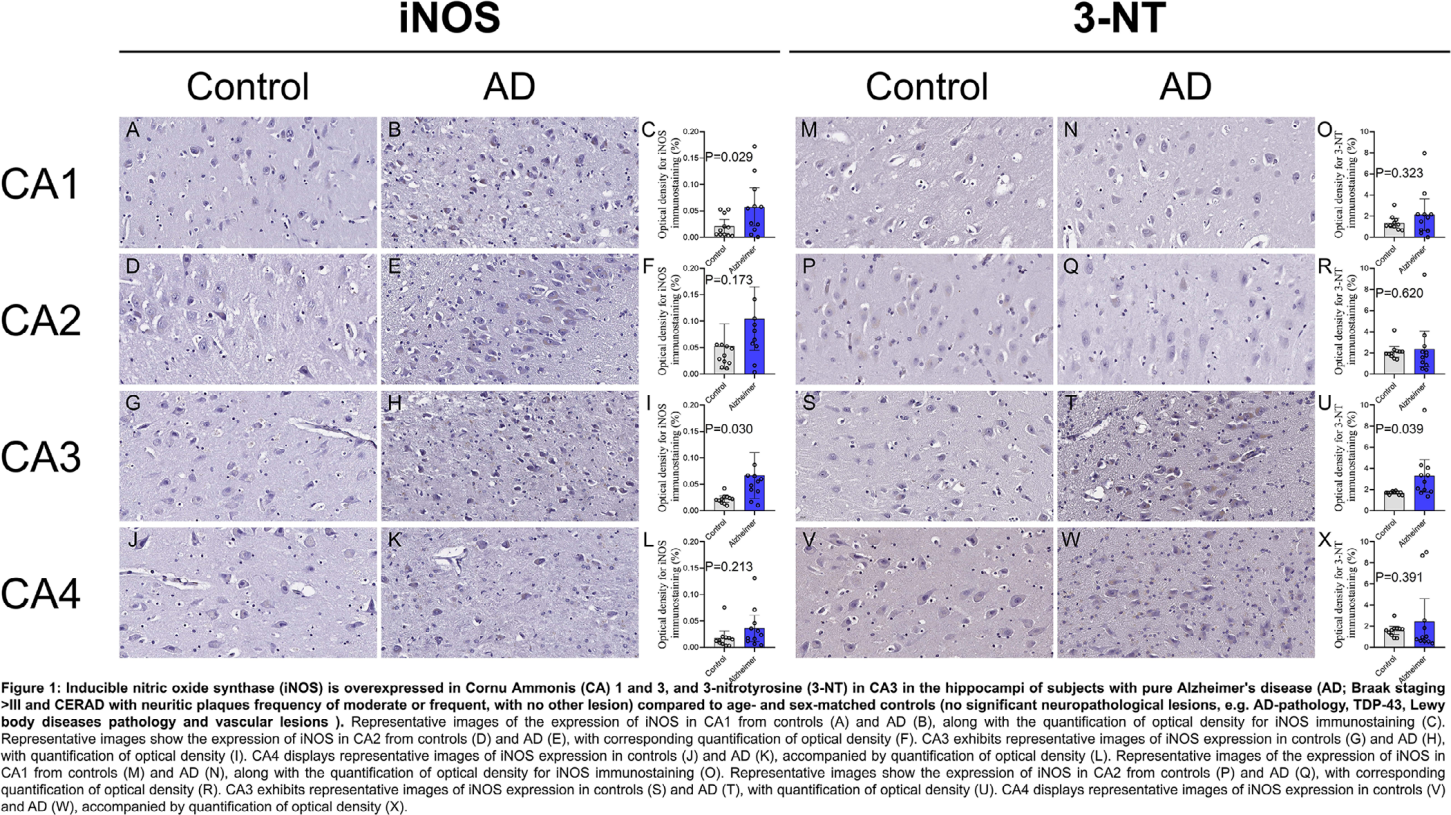# Selective Vulnerability of hippocampi for inducible nitric oxide synthase and 3‐nitrotyrosine in Alzheimer’s disease: A Postmortem Study

**DOI:** 10.1002/alz.085961

**Published:** 2025-01-03

**Authors:** Alberto Fernando Oliveira Justo, Eliana Cristina de Brito Toscano, Vitor Ribeiro Paes, Renata Elaine Paraizo Leite, Carlos Augusto Pasqualucci, Wilson Jacob‐Filho, Ricardo Nitrini, Lea T. Grinberg, Claudia Kimie Suemoto

**Affiliations:** ^1^ Biobank for Aging Studies of the University of São Paulo, São Paulo Brazil; ^2^ University of São Paulo Medical School, São Paulo, São Paulo Brazil; ^3^ Federal University of Juiz de Fora, Medical School, Juiz de Fora Brazil; ^4^ Physiopathology in Aging Laboratory (LIM‐22), Department of Internal Medicine, University of Sao Paulo Medical School, São Paulo, São Paulo Brazil; ^5^ Physiopathology in Aging Laboratory (LIM‐22), University of Sao Paulo Medical School, São Paulo, São Paulo Brazil; ^6^ Biobank for aging studies of the University of São Paulo, São Paulo Brazil; ^7^ University of São Paulo Medical School, São Paulo Brazil; ^8^ Weill Institute for Neurosciences, University of California San Francisco, San Francisco, CA USA; ^9^ Division of Geriatrics, Department of Internal Medicine, University of Sao Paulo Medical School, São Paulo, São Paulo Brazil; ^10^ Division of Geriatrics, University of São Paulo Medical School, São Paulo, São Paulo Brazil

## Abstract

**Background:**

Nitric oxide (NO) is involved in synaptic transmission and cerebral plasticity, playing a role in the memory process. However, in states of brain inflammation, hypoxia, or ischemia, there is induction of inducible nitric oxide synthase (iNOS) expression by astrocytes and pyramidal cells in the brain. Under conditions of chronic activation, there is a decoupling of iNOS dimers, leading to a massive generation of superoxide anion and peroxynitrite, O2.‐ and ONNO‐ respectively, with a significant neurotoxic role by oxidative stress. We aimed to quantify the expression of iNOS and 3‐nitrotyrosine (3‐NT, an oxidative marker of ONNO‐) in the hippocampus of individuals with AD neuropathological changes (ADNC)compared to individuals without AD pathology and investigate the association of iNOS and 3‐NT expression with cognition abilities and AD pathology.

**Method:**

We evaluated the expression of the enzymes iNOS and 3‐NT using immunohistochemistry in the cornu ammonis (CA) and dentate gyrus from hippocampi from subjects with ADNC and the sex‐matched controls. Cognitive abilities were evaluated using the Clinical Dementia Rating (CDR) and AD pathology using Braak staging for neurofibrillary tangles and the Consortium to Establish a Registry for Alzheimer’s Disease (CERAD) for neuritic plaque. We used linear regression to examine the association of iNOS and 3‐NT with cognition and AD pathology.

**Result:**

Sociodemographic and clinical variables were similar between the control and ADNC groups (Table 1). In individuals with ADNC, iNOS was overexpressed in the CA1 (p = 0.029) and CA3 (p = 0.030). Similarly, 3‐NT was overexpressed in CA3 (p = 0.039), shown in Figure 1. No expression was seen in the dentate gyrus for iNOS and 3‐NT. Increased levels of iNOS in CA3 were associated with AD pathology reported in CERAD score (β = 12.44, IC95% = 0.88;24;00, p = 0.036) and Braak staging (β = 25.48, IC95% = 6.12;44.84, p = 0.012), and with cognitive abilities (β = 73.05, IC95% = 29.25;149.90, p = 0.025). Increased 3‐NT levels in CA3 were associated only with cognitive abilities (β = 1.48, IC95% = 0.08;2.87, p = 0.038) (Table 2).

**Conclusion:**

CA3 neurons shows a particular susceptibility to accumulate iNOS and 3‐NT during AD progression. The association between iNOS and functional cognition underscore the role of oxidative stress in AD pathogenesis. Of note, CA2 sector, a resistant region to AD showed no signs of oxidative stress in AD.